# Nosocomial outbreak of neonatal *Salmonella enterica *serotype Enteritidis meningitis in a rural hospital in northern Tanzania

**DOI:** 10.1186/1471-2334-4-35

**Published:** 2004-09-14

**Authors:** Hogne Vaagland, Bjørn Blomberg, Carsten Krüger, Naftali Naman, Roland Jureen, Nina Langeland

**Affiliations:** 1 Department of Otolaryngology/Head & Neck Surgery, Haukeland University Hospital, N-5021 Bergen, Norway; 2 Centre for International Health, University of Bergen, N-5021 Bergen, Norway; 3 Institute of Medicine, University of Bergen, N-5021 Bergen, Norway; 4 Haydom Lutheran Hospital, Mbulu, Tanzania; 5 Deipe Stegge 71, D-48653 Coesfeld, Germany; 6 Department of Medicine, Haukeland University Hospital, N-5021 Bergen, Norway

## Abstract

**Background:**

Clinicians at Haydom Lutheran Hospital, a rural hospital in northern Tanzania noted an unusually high case-fatality rate of pediatric meningitis and suspected an outbreak of an unknown agent or an organism resistant to the empirical therapy.

**Methods:**

We established a provisional microbiology laboratory to investigate the suspected outbreak. Blood and spinal fluid specimens were taken from children below the age of seven years with suspected meningitis. The blood and spinal fluid specimens were inoculated in commercial blood culture bottles and locally prepared Thayer-Martin medium in slanted tubes, respectively. The bacterial isolates were sent to Norway for further investigation, including susceptibility testing and pulsed-field gel-electrophoresis (PFGE).

**Results:**

Among 24 children with suspected meningitis and/or septicemia, five neonates had meningitis caused by *Salmonella enterica *serotype Enteritidis, all of whom died. Two children had *S*. Enteritidis septicemia without meningitis and both survived. Genotyping with PFGE suggested a clonal outbreak. The salmonella strain was resistant to ampicillin and sensitive to gentamicin, the two drugs commonly used to treat neonatal meningitis at the hospital.

**Conclusion:**

The investigation reminds us that nontyphoidal salmonellae can cause meningitis associated with very high case-fatality rates. Resistance to multiple antimicrobial agents increases the risk of treatment failure and may have contributed to the fatal outcome in all of the five patients with salmonella meningitis. The investigation indicated that the outbreak was nosocomial and the outbreak subsided after hygienic measures were instituted. Establishing a provisional microbiological laboratory is a valuable and affordable tool to investigate and control outbreaks even in remote rural areas.

## Background

Nontyphoidal salmonellae are a common cause of food-borne illnesses. In Africa, nontyphoidal salmonellae are the most common cause of bloodstream infections in children younger than five years [1]. While meningitis caused by nontyphoidal salmonellae is uncommon in economically developed countries [2], it is more frequent in tropical countries, particularly in children younger than six months, and associated with higher case-fatality rates than meningitis caused by other bacteria [1,3-5].

At Haydom Lutheran Hospital, a rural hospital in northern Tanzania, clinicians noted an extraordinarily high case-fatality rate (>60%) from pediatric meningitis in the period January 1998 to April 2000. It was suspected that the high case-fatality rate of meningitis was due to an outbreak of an unusual etiological agent, or an organism resistant to the hospital's standard empirical treatment, which was ampicillin and gentamicin for infants (<2 months) and penicillin and chloramphenicol for older children. Thus, we established a provisional microbiology laboratory to identify the causative agents and to facilitate the implementation of effective preventive measures.

## Methods

From a total of 360 children admitted from July to August 2000, blood and/or spinal fluid specimens were collected from 24 children aged one day to six years (median age 23.5 days) with suspected meningitis and/or septicaemia, after careful evaluation by the attending pediatrician. Blood and spinal fluid specimens were inoculated in BBL SeptiChek blood-culture bottles (Becton Dickinson, Sparks, MD USA) and on locally prepared non-selective Thayer-Martin medium in slanted tubes, respectively. All cultures were incubated at 35°C for 5 days and inspected daily for bacterial growth. Positive bacterial specimens were shipped to Institute of Medicine, Haukeland University Hospital, Norway, for further study. The total cost of the laboratory reagents used on site was $475. Positive specimens were identified using standard laboratory methods [6]. The susceptibilities of the isolates to antimicrobial agents were examined by disk diffusion method on PDM medium (AB Biodisk, Solna, Sweden) [7]. The isolates were genotyped with pulsed-field gel electrophoresis (PFGE). Statistics were calculated with Stata 8 for Mac OSX (Stata Corporation, College Station, TX).

## Results

**Figure 1:**
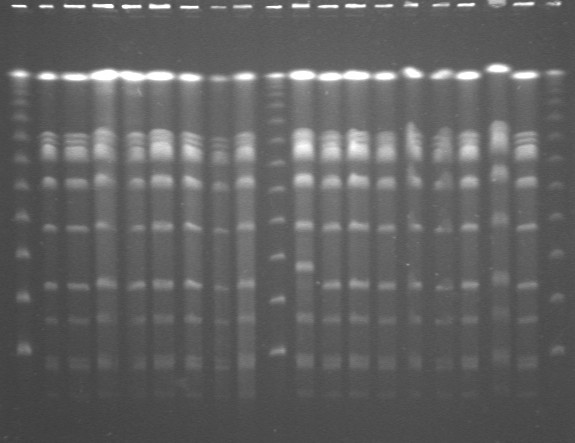
Pulsed-field gel-electrophoresis (PFGE) of digested DNA from 17 isolates of *Salmonella enterica *serotype Enteritidis obtained from seven children with meningitis and/or septicemia. The isolates were obtained from blood cultures of all seven children and spinal fluid culture of four children. Six of the isolates in the gel are duplicate isolates from the same children.

On clinical grounds, twenty-four children were considered to have possible sepsis or meningitis. Blood culture was taken from 23 children, but 13 had received prior antibiotic treatment. Spinal fluid culture was taken from 16 children, of whom ten had received prior antibiotic treatment. Both blood culture and spinal fluid culture was obtained from a total of 15 children. *Salmonella enterica *serotype Enteritidis was isolated from seven patients, of whom four had positive cultures from both blood and spinal fluid and three from blood only. One of the three patients, who had *S*. Enteritidis isolated only from blood culture, had pus in the spinal fluid, and was therefore considered a case of *S*. Enteritidis meningitis, resulting in a total of five cases of *S*. Enteritidis meningitis. One isolate each of *Staphylococcus aureus *and *Streptococcus pyogenes *were isolated from spinal fluid and three isolates of coagulase-negative staphylococci were isolated from blood. Antimicrobial susceptibility testing showed that all the *S*. Enteritidis isolates were resistant to chloramphenicol and ampicillin, intermediate resistant to cefuroxime, and sensitive to gentamicin, cefotaxime and ciprofloxacin. As shown in Figure 1, the PFGE patterns were identical for all the eleven *S*. Enteritidis isolates except one, which differed by only one band (Figure 1 shows a total of 17 strains, 6 of which are duplicate isolates from the same patients). All children with *S*. Enteritidis infections were neonates (median age 15 days, range: 8 to 27 days), whereas those with other infections or negative cultures, on average, were older (median age 4 months, range: 1 day to 6 years). All children with *S*. Enteritidis infections had been delivered at Haydom Lutheran Hospital, and five out of the seven never left the hospital before they became ill. Table 1 shows the characteristics of the patients with *S*. Enteritidis infection. All five children with *S*. Enteritidis meningitis died. The two children with *S*. Enteritidis sepsis survived. Five (29%) of the 17 children without verified *S*. Enteritidis infection also died. Among the 24 children investigated, *S*. Enteritidis meningitis was associated with a relative risk of 3.8 (95% confidence interval 1.8 to 8.1) for fatal outcome.

## Discussion

The finding of an outbreak of bacteremia and meningitis caused by *S*. Enteritidis was not anticipated. Genotyping with PFGE suggested a clonal outbreak. This genotyping information, the susceptibility patterns and the clinical information that all children with *S*. Enteritidis infections were born at the hospital and that the majority never left the hospital before they became ill, strongly suggest that the outbreak was nosocomial. Nontyphoidal salmonella infections are frequently associated with animal reservoirs and infection usually originates from food products. Nosocomial spread of nontyphoidal salmonellae, particularly in neonatal wards, is known from the literature [8]. Neonates are at particular risk of infection because of relatively reduced gastric acidity and peristalsis [1]. In previous nosocomial outbreaks caused by salmonellae, the sources of infection have been related to the use of contaminated medications, diagnostics, blood products, banked human milk, the use of raw eggs or yeast in tube feeding and improper disinfection of devices such as rubber tubes for oropharyngeal suction [8,9]. The exact source of the outbreak at the hospital was not established. Direct food-borne transmission was unlikely, since the neonates at the hospital were fed on breast milk. Possible sources include contaminated instruments, clothes or bathing facilities for the newborn. Spread of infection may have occurred by baby-to-baby transmission or via family members and/or hospital staff. The medical staff at the hospital was informed about the findings, and immediate interventions in the form of hygienic measures were instituted, including the reinforcement of disinfection and hand-washing practices. Data from the hospital annual reports shows that the case-fatality rate from pediatric meningitis dropped from >60% before the intervention to 40% by 2001.

The *S*. Enteritidis strain responsible for the outbreak was resistant to two of the first-line drugs, ampicillin and chloramphenicol, but sensitive to gentamicin. This finding is not surprising, since multi-drug-resistant *S*. Enteritidis has been reported from this region for decades [10]. The children at the hospital were treated with a combination of ampicillin and gentamicin. The high case-fatality rate in these patients implies that *de facto *monotherapy with gentamicin may be suboptimal as treatment for *S*. Enteritidis meningitis considering that gentamicin traverses the blood-brain-barrier poorly, is bound to proteins and inhibited by the acidity in infected cerebrospinal fluid. However, it also reflects the poor prognosis of neonatal *S*. Enteritidis meningitis regardless of therapy. Molyneux reported fatal outcome for 58% of children with meningitis caused by nontyphoidal salmonellae despite routine treatment with chloramphenicol, to which all bacterial isolates were sensitive in vitro [4]. Many authorities recommend third-generation cephalosporins as empirical chemotherapy for meningitis caused by gram-negative bacteria [11], not only because of high bactericidal activity due to low minimum inhibitory concentrations (MICs), but also because they penetrate the blood-brain-barrier better than both gentamicin and chloramphenicol. A third-generation cephalosporin, such as cefotaxime would have been an excellent therapeutic option in this case. However, the price of the newer cephalosporins is often prohibitive in the setting of low-income countries such as Tanzania. Ciprofloxacin is generally not recommended for use in children due to potential adverse effects, but can be resorted to for treatment of life-threatening infections with multidrug-resistant nontyphoidal salmonellae [12]. The standard empirical treatment regimen at the hospital could not be changed due to financial constraints.

Infections caused by nontyphoidal salmonellae in children in Africa are more common during the rainy season and have been associated with malaria, anemia and malnutrition [1]. However, there is considerable overlap between these medical conditions, all of which may be more frequent during the rainy season. This investigation was performed during the dry season. Infections caused by nontyphoidal salmonellae have also been associated with HIV infection [1]. We do not know the HIV status of the children involved in this outbreak, however, in this part of Tanzania, the rate of HIV infection is less than 0.5% [13].

## Conclusions

There are a number of lessons to be learned from this investigation. We are reminded that *S*. Enteritidis can cause meningitis, which carries a very high case-fatality rate [1,3,4]. Our findings support former reports that *S*. Enteritidis can easily spread by nosocomial transmission, particularly in neonatal wards. Resistance to multiple antimicrobial agents increases the risk that empirical therapy will fail, especially in settings where modern cephalosporins are not affordable. Without adequate laboratory facilities, correct diagnosis and treatment of bacterial meningitis and septicemia in children remains a challenge. However, the report also shows that establishing a provisional microbiology laboratory can be a valuable and affordable tool to investigate and curb epidemics even in the setting of remote rural Africa, provided there is a proficient laboratory willing to assist.

## Competing interests

None declared.

## Authors' contributions

HV was the principal investigator, participated in the planning and execution of the work, including performing the on-site microbiological procedures, and analysis of data, and was the main responsible author. BB participated in planning, microbiological investigations in Norway, data analysis and writing. CK and NN undertook clinical investigation and sample collection and participated in the writing. RJ was responsible for the microbiological investigations in Norway and participated in the data analysis and writing. NL was the project coordinator and participated in planning, data analysis and writing. All authors read and approved the final manuscript.

## Pre-publication history

The pre-publication history for this paper can be accessed here:



## Figures and Tables

**Table 1:** Characteristics of neonates with meningitis (n = 5) and sepsis (n = 2) caused by *Salmonella enterica *serovar Enteritidis at Haydom Lutheran Hospital, Northern Tanzania, in July and August 2000

**Patient characteristic**	**Pat. 1**	**Pat. 2**	**Pat. 3**	**Pat. 4**	**Pat. 5**	**Pat. 6**	**Pat. 7**
Sex	F	F	M	F	F	F	M
Mode of delivery	SVD	SVD	Sectio	SVD	SVD	SVD	SVD
Age at onset	8 d	7 d	8 d	15 d	24 d	14 d	13 d
Age at discharge (*death)	20 d	21 d	32 d*	21 d*	29 d*	28 d*	17 d*
Birth weight	2350 g	4000 g	3300 g	2620 g	3600 g	ND	2800 g
Fever	+	-	+	+	+	+	-
Neck stiffness	-	-	-	-	-	-	-
Fits	-	-	-	+	+	+	+
Bulging fontanel	-	-	-	+	+	-	-
*S*. Enteritidis in blood culture	+	+	+	+	+	+	+
*S*. Enteritidis in CSF culture	ND	-	ND	+	+	+	+
CSF WBC	ND	10/μL	Pus	Pus	>3000/μL	ND	32/μL
Clinical diagnosis							
- Meningitis	-	-	+	+	+	+	+
- Septicemia	+	+	+	-	-	+	+
- Malaria	+	+	-	-	+	-	-
Outcome	Alive	Alive	Death	Death	Death	Death	Death

Pat. = Patient, F = Female, M = Male, SVD = Spontaneous vaginal delivery, d = Days, "+" = Present, "-" = Absent, CSF = Cerebrospinal fluid, ND = No data, WBC = White blood cells
